# Transformational Leadership and Employees’ Thriving at Work: The Mediating Roles of Challenge-Hindrance Stressors

**DOI:** 10.3389/fpsyg.2020.01400

**Published:** 2020-06-23

**Authors:** Chun pei Lin, Jialiang Xian, Baixun Li, Haimei Huang

**Affiliations:** ^1^Business Management Research Center, School of Business Administration, Huaqiao University, Quanzhou, China; ^2^Business School, Shantou University, Shantou, China

**Keywords:** transformational leadership, thriving at work, challenge stressors, hindrance stressors, supervisor developmental feedback

## Abstract

Based on previous research, the conceptual model presenting the interaction between transformational leadership, challenge-hindrance stressors and thriving at work was constructed and used to generate the hypotheses for the study. Data were obtained from 542 questionnaires distributed across different organizations. The participants included ordinary employees, grassroots middle and senior managers from China. The major findings are as follows. First, transformational leadership directly is positively related to challenge stressors and thriving at work. Second, challenge stressors are positively relate to thriving at work, while hindrance stressors are negatively relate to thriving at work. Furthermore, challenge stressors mediate the relationship between transformational leadership and thriving at work. Given these findings, the study examined the moderating effect of supervisor developmental feedback on the relationship between transformational leadership and thriving at work. Results reveal that supervisor developmental feedback plays a positive regulatory role between challenge stressors and thriving at work. Additionally, it is shown that the mediating effect of challenge stressors on the relationship between transformational leadership and thriving at work is moderated by supervisor developmental feedback.

## Introduction

Employees are at the forefront of production, R&D, sales, nursing, IT, consulting, and so on, whose ideas and suggestions can directly reflect the crux of the enterprise and can effectively solve various problems of the enterprise. Leaders, as decision-makers and implementers of business operations, will have a profound effect on the employees’ work style and behavior. The biggest challenge in management is how to activate the employees. Therefore, how to stimulate the employees’ work enthusiasm by choosing appropriate leadership styles, to promote employees to learn continuously in the workplace, to generate more innovating behaviors, is a major problem to be solved. However, the high load and fast-paced working conditions, caused by the increasingly fierce labor market competition and increasing work requirements, have brought a lot of work stress to employees and have become a common phenomenon in the workplace (Walumbwa et al., 2018). Based on different attributes of stressors, workplace stressors can be divided into two categories: challenge stressors (positive) and hindrance stressors (negative). Specifically, the former can promote individual development, while the latter can hinder the achievement of goals and personal growth (Cavanaugh et al., 2000; Podsakoff et al., 2007; Culbertson et al., 2009). In addition, direct leadership is the most immediate factor to which employees are exposed (Piccolo and Colquitt, 2006). Interaction between leaders and employees affects employees’ perceptions and evaluations of stress (Culbertson et al., 2010). Accordingly, some questions arise: what leadership styles should leaders employ to avoid hindrance stressors and a negative workplace culture? Likewise, how can challenge stressors be enhanced to generate a positive workplace culture which encourages personal growth and work enthusiasm?

Some literature studies the relationship between thriving at work and leadership style, and found that leadership styles, such as authentic leadership (Leroy et al., 2015), empower leadership (Li et al., 2016), servant leadership (Walumbwa et al., 2018), and family supportive supervisors behaviors (Russo et al., 2018), can significantly affect employees’ thriving at work (Li et al., 2019). The leadership style mainly affects thriving at work by expressing empathy, improving employees’ psychological security, and showing open behavior. For example, authentic leadership can affect the sense of prosperity by providing employees with a healthy and ethical work environment, and it can also affect individual vitality by expressing empathy (Mortier et al., 2016).

Similar to authentic leadership, transformational leadership emphasizes the interactive process between leaders and employees (Dust et al., 2014). It usually advocates learning, encourages innovation, and promotes extensive information sharing (Qu et al., 2015). For example, transformational leadership can enable employees to be satisfied in higher-level internal needs, or stimulate employees’ high-level self-realization needs with organizational vision and work meaning (Bass, 1985). Some scholars have pointed out that transformational leadership can be associated with thriving at work positively (Hildenbrand et al., 2018). However, the role of transformational leadership style is still relatively complicated. The elements of expectation and motivation included in the transformational leadership style will affect employees’ perception of the characteristics of the work situation (Lazarus and Folkman, 1986; Gong et al., 2009). Does transformational leadership play an indirect role in creating thriving at work through challenge or hindrance stressors? What differences exist in the paths of challenge and hindrance stressors?

Finally, external support may also be one of the important adjustment variables that influence the employees’ thriving at work (Wallace et al., 2009). The influence of working environment and emotional factors on employees’ work status is very important (Zhou, 2003). When employees work hard at work but feel little success, the lack of work pleasure and sense of achievement will make employees lose their vitality and weaken their motivation to learn. Senior development feedback can be seen as that superiors provide information that is helpful or valuable to employees’ future learning, development, and improvement (Jaworski and Kohli, 1991). If employees can get instant feedback from superior leaders, whether the work fun and sense of accomplishment derived from instant feedback can adjust employees’ stress perception and make employees more likely to be in a strong and active working state? That is, does supervisor developmental feedback play a role in this moderating mechanism?

We build a conceptual model to explore the relationship among transformational leadership, challenge-hindrance stressors, thriving at work, and senior development feedback. First, we use transformational leadership as the independent variable and thriving at work as the dependent variable to clarify the relationship between transformational leadership and thriving at work. It will help us expand the research on antecedent variables of thriving at work. Second, based on the theory of social information processing and stress-cognitive interaction theory, we explored the intermediary role of challenge-hindrance stressors in the above relationship. By exploring the mediating effect of work stress, we can open the black box in which transformational leadership affects employees’ thriving at work. Finally, by take superior development feedback as a moderating variable, this study further explores the relationship between transformational leadership style and thriving at work through the use of a moderated mediation model.

## Theoretical Analysis and Research Hypotheses

### Literature Review

According to the socially embedded model of thriving at work, whether it is the characteristics of the work situation, or the individual’s active behavior (i.e., the individual’s active and purposeful behavior at work), or basic psychological needs, all of them are related to thriving at work (Spreitzer et al., 2005). Therefore, we attempt to study the relationship between transformational leadership and thriving at work, with consideration of the mediating roles of challenge-hindrance stressors.

First, from the work situation characteristics, leadership style can be used as a key element of a workplace. Transformational leadership is defined as the interactive process between leaders and employees, and emphasizes that leaders should propose higher levels of ideals, beliefs, and values, and thereby enhances the subordinates’ consciousness, so that employees can be satisfied in the higher levels of internal needs (Burns, 2004). The existing articles believe that transformational leadership encourages subordinates to sacrifice their personal interests for the benefit of the organization, by giving more meaning to their work, inspiring their subordinates’ high-level needs, and creating an atmosphere of mutual trust (Bass, 1985). In order to meet the leaders’ expectations, employees may continue to improve their abilities, generate more organizational citizenship behaviors, and stimulate creative thinking (Podsakoff et al., 2000; Judge and Piccolo, 2004).

Second, from the perspective of employees’ behavior and psychology, transformational leadership can also be related to employees’ thriving at work by affecting employees’ perception of stress on work events or situations (Cavanaugh et al., 2000). The stress caused by challenging sources can be overcome by the individual, which can be positively related to individual performance and growth, such as workload, time urgency, work scope and responsibilities, work complexity, and so on Lepine et al. (2005). The individuals believe that once they overcome the challenge stressors, they will get benefits and rewards in terms of job performance, promotion and future growth, so they will adopt active strategies (Webster et al., 2011). In contrast, the stress, caused by hindrance sources, is difficult for individuals to overcome. It will hinder the achievement of individual work goals and career development, such as organizational politics, role ambiguity and conflict, bureaucratic procedures, and job insecurity and so on Boswell et al. (2004). The individuals believe that they will not obtain any benefits and rewards in the foreseeable future, and then adopt negative strategies such as retreating or leaving.

According to social information processing theory and stress cognition interaction theory, the leaders, as the most significant and most relevant source of social information in the workplace, can affect employees’ cognition, evaluation and response to stress (Lazarus and Folkman, 1986). Employee’s perception of different stressors will make employees behave differently, and have different psychological satisfaction, which directly affects the experience level of employees’ thriving at work. Therefore, it can be seen that both transformational leadership and employees’ perception of work stress may become motivations for inspiring employees’ thriving at work.

Third, whether faced with positive stress or negative stress, the individual needs to pay a lot of effort to deal with it, which will consume a lot of emotional resources. The relevant resources available to the individual at work can reduce the adverse effects caused by excessive work requirements (Wilmar et al., 2004), and the supervisor developmental feedback is a kind of work resource given to the individual by the leader, which can effectively adjust the psychological state of employees and help employees to experience the positive significance of work (Jaworski and Kohli, 1991), and can be referred to the extent to which supervisors provide employees with helpful and useful information that enables employees to learn, develop, and make improvements (Zhou, 2003).

This feedback method has the following three characteristics: (1) The feedback source is superior. Supervisors urge or encourage employees to work actively and improve organizational performance by providing suggestions with reference value for employees. (2) The feedback content is relatively rich. It can be based on the problems or doubts in the work, which can be related to employee behavior. (3) The recipient of the feedback is an employee. After receiving the feedback, the employees make corresponding adjustments to meet the work requirements of the superior. Although the transformational leadership can shape a specific situation mechanism for employees, due to the different developmental feedback received by employees, there may be differences in the relationship among work stress perception, individual emotions and cognitive status. Therefore, it is necessary to incorporate supervisor developmental feedback into the model to explore its moderating role among transformational leadership, challenge-hindrance stressors, and thriving at work.

**FIGURE 1:**
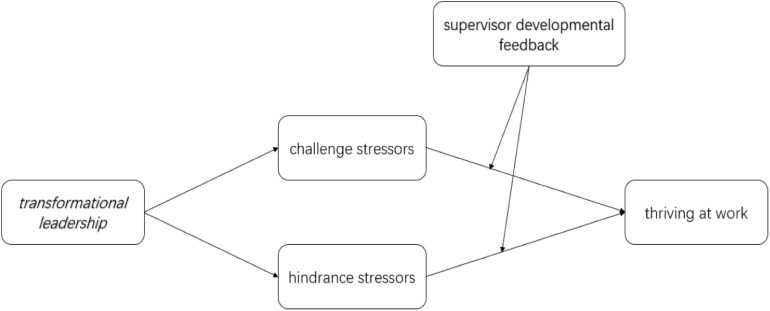
Research Model.

To sum up, by taking transformational leadership as the independent variable, challenge-hindrance stressors as the intermediary variable, thriving at work as the dependent variable, and supervisor developmental feedback as the adjustment variable, we construct the research model which is shown in Figure 1 below.

### Transformational Leadership and Employee Stressors

As the most significant and relevant source of social information in the workplace, leaders can significantly impact employees’ perceptions of job characteristics (Piccolo and Colquitt, 2006). Similarly, from the perspective of leaders’ management of workplace meaning, leaders structure subordinates’ daily work environment and set reference points to help them better understand their daily tasks (Smircich and Morgan, 1982). Furthermore, according to the theory of stress-cognitive interaction, employees evaluate different stressors according to the characteristics of the working environment and their own resource status. Organizational vision, role models, expectations, and care included in the transformational leadership will influence the construction of the work environment, the judgment of loss of resources, and process of stressors evaluation (Sosik et al., 2004). Given this, the stressors of employees which increased by the transformational leadership may divided into two sides—challenge and hindrance Stressors.

On the one hand, the transformational leadership helps to increase employees’ challenge stressors and enables them to perceive high-level job requirements including workload, work complexity, and urgency of a task. Simultaneously, employees are encouraged through the use of appropriate work resources such as empowerment, mentoring, care, and intellectual inspiration (Lazarus and Folkman, 1986). For example, the transformational leadership allows employees to recognize and adhere to a common organizational vision as well as encourages their willingness toward greater effort in the workplace (Piccolo and Colquitt, 2006). In the process of mobilizing employees’ efforts, transformational leadership also increases employees’ opportunities for promotion. Second, transformational leadership emphasizes setting challenging goals for employees, giving employees greater work responsibility and autonomy, helping them improve their level of demand and intrinsic motivation, and motivating them to pursue higher levels of self-realization (Brief and Weiss, 2002; Gong et al., 2009). With such leadership, employees are able to gain a sense of autonomy, satisfaction and accomplishment. These potential rewards (e.g., promotion opportunities, personal growth, and self-realization) can offer compensation for a loss in employees’ resources (Crawford et al., 2010), prompting employees to evaluate the stress they face as benign and positive (Lepine et al., 2005) as well as helping them realize the work in which they are engaged is important and positively challenging. Therefore, employees exposed to the transformational leadership tend to make assessments on their work situations based on challenge stressors.

On the one hand, the transformational leadership is likely to increase employees’ challenge stressors and enables them to perceive high-level job requirements including workload, work complexity, and task urgency. Specifically, in the process of interaction between leaders and subordinates, employees are in a more passive position and set to work in roles which are moldable, positively responsive to leaders, and subject to the abandoning of self-interest (Tourish, 2013). Moreover, employees are prone to over-reliance and subservience when in a situation with greater uncertainty, which may increase employees’ negative stress perceptions. Additionally, transformational leadership often encourages employees to work harder and show more organizational citizenship behaviors through affecting their emotions, motivations and values (Wang et al., 2005; Dust et al., 2014). However, excessive organizational citizenship behaviors can lead to behavioral conflicts within employees’ roles, which inevitably puts stress on staff (Tepper et al., 2001). Second, transformational leadership often creates high expectations for employees and tends to allow employees to change their preconceptions regarding existing work and to face new work challenges. Compared with a stable and sustainable work environment, this kind of work situation greatly increases the possibility of creating more pressure due to work uncertainty. Finally, transformational leaders seek to create an organizational vision which inspires employees to constantly challenge themselves and achieve innovation. In this process, employees with influence and an ability to adapt are more likely to enter decision-making processes and to make self-interested assessments for supervisor behaviors, thereby increasing their perceptions of stress caused by organizational politics. It should also be pointed out that the high workload instigated by the transformational leadership may also lead to the formation of employees’ organizational political perceptions (Ferris et al., 1989). Based on these insights, we propose the following hypotheses:

H1: The transformational leadership has a positive relationship with challenge stressors.
H2: The transformational leadership has a positive relationship with hindrance stressors.

### Transformational Leadership and Thriving at Work

Generally speaking, the most direct contextual impact factors employees are exposed to are direct leaders, whose leadership style plays an important role in employee performance and is considered a major source of employees’ positive or negative emotional experiences (Brief and Weiss, 2002; Bono and Ilies, 2006; Dasborough and Ashkanasy, 2016). Transformational leadership can evoke or trigger followers’ similar emotions by releasing energetic and positive emotions. More importantly, the transformational leadership, as a type of emotional leadership style with a strong emotional and spiritual component, can stimulate subordinates’ intrinsic motivation, enthusiasm for work, recognition and acceptance of organizational vision and goals. The key behavioral elements of the transformational leadership can directly or indirectly affect employees’ work dynamism. Transformational leadership focuses on passing the vitality inherent in this style to employees through demonstration. Employees who absorb this vitality often have greater energy and achieve high-performance targets which exceed expectations (Shin and Zhou, 2003). Ultimately, transformational leadership can stimulate employees’ creative thinking (Ronit et al., 2003; Noruzy et al., 2013) and support them in adopting new ideas and new methods in their work (Lazarus and Folkman, 1986), which in turn significantly predicts employees’ levels of work vitality (Shirom, 2003). Personal care for employees also helps to improve their internal motivation levels and creates greater enthusiasm and engagement toward the work they perform (Lazarus and Folkman, 1986; Mittal and Dhar, 2015).

Leadership is a process which promotes individual and collective efforts toward learning to accomplish organizational common goals. The transformational leadership can influence employees’ learning experiences by focusing on helping them to absorb a proactive learning atmosphere, in turn affecting their learning commitments and need for growth. In an uncertain environment, a clear and exciting organizational vision can give employees motivation and direction toward learning. If employees do not agree with and commit to the developmental direction determined by the organization, they will lack motivation toward learning and growth (Mckee, 2010). Transformational leadership can influence and drive subordinates to work hard for a common organizational vision, promote trust and cooperation between employees and teams, and form a cohesive and centripetal learning team to strengthen employees’ learning motivation (Han et al., 2016). Accordingly, transformational leadership tends to cultivate knowledge innovators, often encourages employees to innovate and challenge themselves and also instills new ideas into employees, all of which transform employees’ mental models and inspires them to adopt innovative ways toward problem-solving—this promotes active learning (Piccolo and Colquitt, 2006; Dust et al., 2014). Additionally, transformational leadership embodies the characteristics of granting employees greater autonomy and supporting them to use their acquired knowledge and experience to enhance themselves (Dvir et al., 2002). Given this, we propose the following hypothesis:

*H3: The transformational leadership has a positive relationship with employees*’ thriving at work.

### Challenge-Hindrance Stressors and Thriving at Work

When individuals face challenge stressors, work requirements such as workload and job responsibilities can bring potential benefits to compensate for the loss of their resources and individuals may show positive work attitudes and behavioral tendencies. In the face of hindrance stressors, organizational politics and role ambiguity are likely to consume individual energy resources and will not bring returns, resulting in individuals with negative work attitudes and behavioral tendencies (Podsakoff et al., 2007; Prem et al., 2017). More specifically, the theory of stress-cognitive interaction claims employees will initiate a cognitive evaluation process in their interaction with the surrounding environment (Lepine et al., 2005). Employees will take further steps to respond to the events they encounter based on the results of the primary evaluation (i.e., unrelated, benign or stressful), which in turn affects their mood and behavior. If employees make positive evaluations regarding work events, they will adopt a positive problem-solving approach and make efforts toward gaining new knowledge and the skills necessary to better resolve work issues. Conversely, if an employee makes a negative evaluation, they will choose a negative approach.

According to self-determination theory, when individuals face challenges, the potential associated with self-determination can lead them to engage in an activity or task of interest and benefit to the development of their ability (Boswell et al., 2004). In turn, the individual is more engaged in the activity and obtains a sense of satisfaction and accomplishment from it. This, however, will not be experienced when an individual is faced with a hindrance stressors (Ryan and Deci, 2008). Furthermore, challenge stressors are positive in terms of employee performance and self-growth and can stimulate individual motivation and positive emotions such as happiness and satisfaction. Consequently, employees are in an activated working state when engaged in a challenge-stressors approach. In contrast, hindrance stressors related negatively to employees’ career development and will generate in employees negative emotions such as anxiety, sadness and fear. This negative psychological state is not conducive to stimulating employees’ vitality at work. Based on these insights, we propose the following hypotheses:

*H4: Challenge stressors have a positive relationship with employees*’ *thriving at work.*
*H5: Hindrance stressors have a negative relationship with employees*’ *thriving at work.*

### Mediating Effect of Challenge-Hindrance Stressors Between the Transformative Leadership Style and Thriving at Work

Challenge and hindrance stressors are analogous to perceptions of stimuli contained in the work situation and are an important link between the work situation factor and employees’ work attitudes and behaviors. Relevant empirical studies show that employees’ evaluation of work situations is an important antecedent variable which affects employees’ perceptions of challenge-hindrance stressors (Boswell et al., 2004; Lepine et al., 2005; LePine et al., 2016). Simultaneously, challenge and hindrance stressors are also important variables which affect employees’ work attitudes and behaviors. As an important factor in the work situation, leadership style often affects employees’ perceptions and evaluation of job characteristics and, thus, can be related to employees’ working status (Culbertson et al., 2010). At present, there exists limited research on the relationship between transformational leadership and employees’ thriving at work. Therefore, the current study addresses this topic in combination with self-determination theory.

Self-determination theory emphasizes the relationship between environmental factors and individual initiative (Gagné and Deci, 2005). It claims the external environment is capable of strengthening individual internal motivation and promoting the internalization of external motivation by satisfying individuals’ basic psychological needs (e.g., autonomy, competency and attribution needs). The satisfaction of basic psychological needs is a key factor in promoting employees’ thriving at work (Prem et al., 2017). When employees’ work environment allows for the realization of their basic psychological needs, individual vitality and internal motivation are enhanced (Ryan and Deci, 2008); employees are more likely, then, to experience thriving at work. On the one hand, the transformational leadership allows employees to experience challenge stressors by creating challenging working conditions and avenues encouraging autonomy (Dust et al., 2014). This allows employees to take positive actions on their own to achieve satisfactory results in challenging work, as well as to meet their autonomy and competency needs. Therefore, the transformational leadership is capable of promoting improvements to thriving at work. On the other hand, the transformational leadership may make employees feel there are organizational politics and work uncertainty in their work situation (Lepine et al., 2004; Mckee, 2010; Han et al., 2016), thus leading to the experience of hindrance stressors. Furthermore, as negative emotions and coping styles fail to meet employees’ basic psychological needs, employees’ vitality is hindered and they experience difficulties entering a thriving state.

The current study argues that the transformational leadership is capable of enhancing employees’ basic psychological needs by promoting challenge stressors and that, in turn, this increases employees’ thriving at work. More specifically, challenge stressors mediate the role of the transformational leadership in increasing employees’ thriving at work, where both the role of the transformational leadership in challenge stressors and the relationship between challenge stressors and employees’ thriving at work are positive. Simultaneously, the transformational leadership may increase hindrance stressors which hinder employees’ basic psychological needs and, thus, inhibit employees’ thriving at work. More specifically, hindrance Stressors mediate the role of the transformational leadership in increasing employees’ thriving at work, where the role of the transformational leadership in terms of hindrance stressors is positive, and the relationship between hindrance stressors and employees’ thriving at work is negative. Give this, we propose the following hypotheses:

*H6: Challenge stressors mediate the role of the transformational leadership in increasing employees*’ *thriving at work.*
*H7: Hindrance stressors mediate the role of the transformational leadership in increasing employees*’ *thriving at work.*

### Moderating Effect of Supervisor Developmental Feedback

Supervisor developmental feedback focuses on subordinates’ future development and improvement of their abilities, emphasizing that supervisors should provide employees with targeted, future-based feedback which will help them learn, develop and improve (Jing, 2003; Bono and Ilies, 2006). Supervisor developmental feedback is a special supportive work resource from supervisors and has a higher perceived value compared with peer-to-peer feedback (Ashford and Tsui, 1991). Whether facing challenges or hindrance stressors, access to such resources is important for employees. According to resource conservation theory, employees need to make efforts to cope with challenge and hindrance stressors to complete their work tasks. Furthermore, the more effort made, the more likely employees will experience emotional exhaustion if more work resources are lost (Schaufeli and Bakker, 2004; Jiang et al., 2014). Therefore, the impact of supervisor developmental feedback on the relationship between challenge-hindrance stressors and thriving at work is particularly noteworthy.

Employees will evaluate stress events in their work situations according to their own resources and ability status. Employees who have obtained different levels of developmental feedback in turn experience different perceptions of stress and coping styles as well as different psychological experiences. When employees receive greater supervisor developmental feedback, the resources available to deal with challenge-hindrance stressors increase, employees’ interest in the task itself is enhanced, and work processes are more enjoyable (Ashford and Tsui, 1991). Consequently, it is beneficial to stimulate employees’ internal motivations as well as guide their learning behaviors (Shalley and Perry-Smith, 2001). The accompanying increase in knowledge and skill level can generate positive emotions among employees and, thus, meet employees’ personal growth needs. As a result, challenge stressors to a greater extent will affect employees’ mental states, while the negative correlation of hindrance stressors with employees is weakened. In contrast, employees who receive less developmental feedback from their supervisors are more likely to fall into a negative state due to the loss of their own resources. Therefore, hindrance stressors can affect employees’ mental states to a greater extent, and employees’ incentives in terms of challenge stressors become less sensitive.

The current study proposes that supervisor development feedback adjusts the relationship between challenge-hindrance stressors and thriving at work. Specifically, compared with the case in which supervisor development feedback is lower, when supervisor developmental feedback is higher, employees obtain a greater number of working resources and external affirmation, both of which compensate for the consumption of employees’ emotional resources owing to challenge-hindrance stressors. Therefore, employees can be more proactive in assessing and responding to stress based on their resources and capabilities. In turn, this enhances the positive relationship among challenge stressors, employees’ internal motivations and mental states. At the same time, this weakens the negative relationship among hindrance stressors, employees’ internal motivations and mental states. Based on these insights, we propose the following hypotheses:

H8: Supervisor developmental feedback positively moderates the relationship between challenge stressors and thriving at work.
H9: Supervisor developmental feedback negatively moderates the relationship between hindrance stressors and thriving at work.

As mentioned previously, H6 and H7 suggest challenge-hindrance stressors play a mediating role between the transformational leadership and thriving at work. Additionally, H8 and H9 suggest that employees, due to obtaining different levels of supervisor developmental feedback, will have different levels of sensitivity to challenge and hindrance stressors. Furthermore, there exist differences in the relationship between challenge-hindrance stressors and employees’ thriving at work. In the current study, we propose a moderated mediation model in which the mediating role of challenge-hindrance stressors is dependent on the level of supervisor developmental feedback.

According to reciprocal norms of social exchange theory, supervisors expect subordinates to make corresponding returns when providing supporting resources (Wilson et al., 2010). The transformational leadership implies high-performance and creativity expectations for subordinates in a one-way resource supply process (Qu et al., 2015). Correspondingly, employees affected by transformational leadership tend to generate more return obligations and assume more work responsibilities (Dulebohn et al., 2012). However, supervisor developmental feedback is information-based feedback which emphasizes individual performance results, includes fewer return expectations and obligations, and does not impose hard requirements on the specific content and operation of work (Ashford and Tsui, 1991). Accordingly, it allows employees to gain a greater number of working resources, in turn creating a greater inclination toward evaluating stress events as benign and maintaining a thriving-at-work status (Xanthopoulou et al., 2009). Therefore, although transformational leadership provides support and encouragement to employees, due to differences in supervisor developmental feedback, employees still experience differences in stressors perceptions, which in turn affect their thriving at work. Specifically, when supervisor developmental feedback is higher, employees are more confident in their resource status following the influence of transformational leadership and tend to consider stress events as challenge stressors, thus experiencing greater thriving at work. In contrast, when supervisor developmental feedback is lower, the transformational leadership has little influence on employees’ thriving at work through challenge stressors; however, hindrance stressors have a greater influence on employees’ thriving at work. Given these insights and corresponding hypothesis, we consider that supervisor developmental feedback has a moderating impact on the mediating role of challenge-hindrance stressors. When supervisor development feedback is higher, challenge stressors have a stronger positive mediating effect and hindrance stressors have a weaker negative mediating effect. Accordingly, we propose the following hypotheses:

H10: Supervisor developmental feedback positively moderates the mediating role of challenge stressors.
H11: Supervisor developmental feedback negatively moderates the mediating role of hindrance stressors.

**FIGURE 2:**
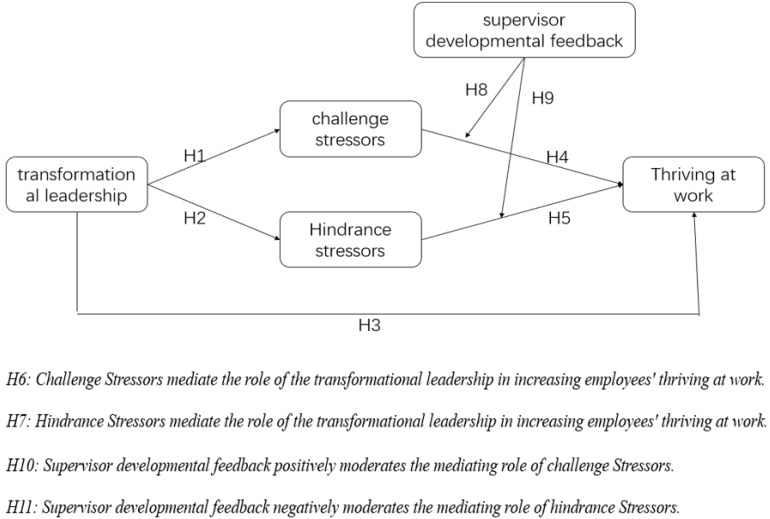
Hypotheses Model.

Figure 2 shows all the hypotheses presented in the article.

## Research Design

### Variable Measurement

In the current study, we used a revised scale based on Podsakoff et al. (1990), Farh and Cheng (2000) to measure the transformational leadership. This 23-item scale reflects the six key behavioral elements of transformational leadership, including vision motivation, behavioral demonstration, promotion of collaboration, high-performance expectations, personalized care, and intellectual stimulation. To measure transformational leadership, these items included statements such as “draw a desirable future for our team,” “guide us through practical actions, instead of simply telling us what to do,” “work hard to promote cooperation between teams,” “clearly express great expectations for us,” “consider my personal needs,” and “encourage me to think about the original problem in a new way.”

Additionally, we used a scale developed by Cavanaugh et al. (2000) to measure challenge and hindrance stressors. This 11-item scale is divided into two categories, where six items measure the challenge stressors and five items measure the hindrance stressors. The scale reflects individuals’ perceptions of different types of stress at work, including that associated with workload, time stress, the scope of work, organizational politics, role ambiguity, and job insecurity. The items are represented by statements such as “I have a lot of stress on the excessive projects I have undertaken,” “I spent too much time on my work and thereby put a lot of stress on me,” and “focusing on identity background and relationships rather than performance in the firm, makes me feel stressed.”

We also used a scale developed by Porath et al. (2012) to measure employees’ thriving at work. This 10-item scale reflects two dimensions of employees’ thriving at work: employees’ vitality and employees’ learning experiences at work. The items were represented by statements such as “I am always energetic at work,” “I am always alert and awake at work,” and “I learn more and more things as my working hours increases.”

To measure supervisor developmental feedback in the current study, we used a scale developed by Zhou (as cited in Ashford and Tsui, 1991). This is a 3-item scale including statements such as “feedback from the direct supervisor is mainly to help me to learn and make progress,” “direct supervisors often give me some feedback suggestions for self-improvement,” and “direct supervisors often give me some information that is conducive to improving work performance.”

The above scales all use Likert six-point scale, where “0” = strongly disagree, “1” = disagree, “3” = slightly disagree, “4” = slightly agree, “5” = agree, and “6” = strongly agree. Finally, to control the impact of other variables in the analysis, based on the previous literature, this study treated gender, age, education, working years, job rank, enterprise type and industry as control variables.

### Pre-Test

In order to improve the reliability and validity of the data, before conducting a large sample survey, this article first implemented a small sample pre-experiment. The pre-experiment survey samples include online and offline. The offline samples are mainly from Fuzhou, Xiamen, Quanzhou, and other local enterprises and institutions. The employees of each enterprise and institution are required to fill in the site and the questionnaire is withdrawn on the spot. We also conducted the survey by the way of publishing the questionnaire on a web platform which is called as “Questionnaire Star” in China. The sample source includes internal employees of enterprises in Fujian, Zhejiang, Shanghai, Hubei, Guangdong, and Jiangsu in China.

In this article, the internal consistency coefficient was used to evaluate the reliability of the pre-test questionnaire. By referring to Cronbach et al. (1951), the item should be deleted if the CITC coefficient is less than 0.5, and the Cronbach’s α coefficient increases significantly after deleting this item. As shown in Table 1, the Cronbach’s α-values of all sub-scales are above 0.8. Especially, the CITC coefficients of TF16, V3 and L4 are less than 0.5, and the CITC of other measurement items are all greater than 0.6. After deleting the items of TF16, V3, and L4, the Cronbach’sα of the subscale increased significantly, and the Cronbach’s α value of the total scale is 0.929.

**TABLE 1:** Reliability analysis for the pre-test.

Variables	Item	CITC	Value of CA after excluding TF16, V3, and L4	Value of CA for total scale and subscale
Visioning and inspiring	TF1	0.719	0.901	0.910
	TF2	0.716	0.903	
	TF3	0.879	0.869	
	TF4	0.757	0.894	
	TF5	0.803	0.884	
Role models	TF6	0.791	0.875	0.902
	TF7	0.812	0.856	
	TF8	0.821	0.847	
Promoting cooperation	TF9	0.756	0.868	0.893
	TF10	0.720	0.880	
	TF11	0.811	0.844	
	TF12	0.781	0.857	
Expecting high performance	TF13	0.708	0.861	0.872
	TF14	0.777	0.800	
	TF15	0.781	0.795	
Individualized care	TF16	0.347 (unreasonable)	0.875 (unreasonable)	Initial value of CA:0.800
	TF17	0.791	0.673	Final value of CA:0.875
	TF18	0.668	0.724	
	TF19	0.689	0.711	
Intellectual stimulation	TF20	0.828	0.931	0.940
	TF21	0.854	0.923	
	TF22	0.898	0.908	
	TF23	0.850	0.924	
Challenge stressors	CS1	0.816	0.925	0.936
	CS2	0.823	0.923	
	CS3	0.859	0.918	
	CS4	0.828	0.922	
	CS5	0.747	0.932	
	CS6	0.801	0.925	
Hindrance stressors	HS1	0.674	0.837	0.862
	HS2	0.692	0.831	
	HS3	0.644	0.843	
	HS4	0.652	0.841	
	HS5	0.751	0.816	
Vitality	V1	0.845	0.803	Initial value of CA:0.870
	V2	0.851	0.804	Final value of CA:0.931
	V3	0.273 (unreasonable)	0.931 (unreasonable)	
	V4	0.763	0.826	
	V5	0.791	0.818	
Learning	L1	0.776	0.807	Initial value of CA:0.860
	L2	0.776	0.806	Final value of CA:0.917
	L3	0.804	0.805	
	L4	0.408 (unreasonable)	0.917 (unreasonable)	
	L5	0.745	0.814	
Supervisor developmental feedback	SDF1	0.764	0.908	0.907
	SDF2	0.819	0.862	
	SDF3	0.861	0.825	
Value of CA for total scale after excluding TF16, V3, and L4				0.929

We also find that after deleting the items of TF16, V3 and L4, the test results of the pre-survey sample data also show that the KMO value of each scale in the questionnaire is within the acceptable range of 0.731 and 0.892 (*p* = 0.000, *p* < 0.05), which is suitable for factor analysis. On this basis, an exploratory factor analysis is performed to extract factors with eigenvalues greater than 1 and determine the factor loading of each measurement item. We find that the scale of transformational leadership extracted 6 factors through the maximum variance rotation method, and each factor load was above 0.5, and its cumulative variance interpretation rate was 82.036%. The scale of challenge-hindrance Stressors extracted two factors through the maximum variance rotation method, and the factor loads of each item were above 0.7, and the cumulative variance interpretation rate was 71.611%. The scale of thriving at work has extracted two factors through the maximum variance rotation method, namely vitality and learning, each factor load is above 0.7, and its cumulative variance interpretation rate is 81.971%. The scale of supervisor developmental feedback has extracted one factor through the maximum variance rotation method. The factor load of each item is above 0.8, and the cumulative variance interpretation rate is 84.359%. Each part of the scale has good reliability and validity, and the data collected by the questionnaire survey has good reliability and validity. Therefore, the item TF16, V3, and L4 are excluded.

### Sample Distribution

This study used questionnaires distributed to employees of various organizations for data collection. We entrusted a professional organization to distribute questionnaires among staff working in different industries. The study began in March 2016 and ended in September 2016. It spanned 6 months and a total of 1092 questionnaires were collected. 550 Invalid questionnaires were excluded, 542 valid questionnaires are remained. A demographic analysis of the valid samples found that the proportion of males (41.7%) was slightly lower than that of females (58.3%). The proportion of 25-years-old and younger, 26–35-years-old, 36–45-years-old, and 46-years-old and above accounted for 36.9%, 55.4%, 7.0%, and 0.7%, respectively. The proportion of high school and below, junior college, undergraduate and postgraduate degrees accounted for 6.3%, 14.4%, 62.7%, and 16.6%, respectively. The working years’ proportion of two years and below, 3–5 years, 6–10 years, and 11 years and above accounted for 33.9%, 38.7%, 20.8%, and 6.5%, respectively. The proportion of employees, grassroots managers, middle managers and senior managers accounted for 61.6%, 25.1%, 10.7%, and 2.6%, respectively. In terms of the organizational characteristics of the surveyed enterprises, the proportion of state-owned enterprises, private enterprises and foreign-funded enterprises accounted for 38%, 45.4% and 16.6%, respectively. The proportion of manufacturing, computer services and software, finance, real estate and construction, culture and entertainment, transportation/storage and postal services, and other industries accounted for 24.7%, 16.1%, 16.6%, 7.0%, 4.6%, 5.35%, and 25.6%, respectively.

To check for non-response bias, we compared early respondents (i.e., the first third of the responses received) responded differently from late respondents (i.e., the last third of the responses received) (Armstrong and Overton, 1977). Multivariate *t*-tests with Transformational leadership, thriving at work, challenge stressors, hindrance stressors and supervisor developmental feedback showed no significant difference between early and late respondents. The detail results are shown in Table 2.

**TABLE 2:** *T*-test of independent samples.

		Levene’s test for equality of variances		*T*-test for equality of means		
		*F*	Sig.	*t*	*df*	Sig. (2-tailed)
Gender	Equal variances assumed	4.105	0.043	1.045	540	0.297
	Equal variances not assumed			1.045	539.876	0.297
Education	Equal variances assumed	0.352	0.553	−1.622	540	0.105
	Equal variances not assumed			−1.622	539.98	0.105
Working years	Equal variances assumed	4.579	0.033	−0.621	540	0.535
	Equal variances not assumed			−0.621	537.936	0.535
Job rank	Equal variances assumed	0.761	0.384	−1.641	540	0.101
	Equal variances not assumed			−1.641	531.32	0.101

### Research Method

This article mainly adopts the empirical research method of questionnaire survey, and revises the original questionnaire according to the specific conditions of the survey object and the research purpose. First, a small sample pre-experiment is implemented, and the questionnaire is further modified by analyzing the pre-experiment data. Then, on this basis, a large sample questionnaire survey was conducted to collect relevant data. In statistical analysis, this article uses SPSS 20.0 software to perform descriptive statistical analysis, reliability and validity test and regression analysis on sample data. At the same time, this article uses AMOS 22.0 software for confirmatory factor analysis, and evaluates the fitting of the measurement model and the structural model according to the model fitting index. In the main effect and intermediary effect, we mainly use structural equation modeling to analyze the transformational leadership and challenge-hindrance stressors on thriving at work, and examine the intermediary role of challenge-hindrance stressors. When verifying the moderating effect, by referring to Toothaker (1991), we mainly used hierarchical regression for analysis and hypothesis testing, and conducted a moderating intermediary effect test on the model through the sequential testing method.

## Empirical Results and Analysis

### Reliability and Validity Analysis

The reliability of the questionnaire was evaluated by calculating the correlation coefficient (CITC) and the internal consistency coefficient (Cronbach’s alpha). The CITC of all items was found to be above 0.771. The Cronbach’s alpha coefficients of transformational leadership, challenge stressors, hindrance stressors, thriving at work, and supervisor developmental feedback were 0.961, 0.907, 0.918, 0.890, and 0.936, respectively. The alpha coefficient also reached 0.941, demonstrating the strong reliability of the scale in total. The measurement items used in the current study were adjusted through pre-testing and were found to have strong validity. Therefore, to obtain a relatively simple model and to create more stable parameter estimation, measurement items were divided into dimensions and averaged for packaging. We then used the confirmatory factor analysis technique to evaluate the discriminant validity among the variables. Table 3 shows the comparison results of five measurement models. From Table 3, it can be seen that the six-factor model has an acceptable goodness of fit compared with alternative models, as χ^2^/df = 2.413, CFI = 0.953, GFI = 0.904, TLI = 0.947, IFI = 0.953, RMSEA = 0.051 in the six-factor model. Therefore, the six factors involved in the analysis had good discriminant validity and accurately represented the six different constructs.

**TABLE 3:** Measurement model comparison.

Model	χ ^2^	df	χ ^2^/df	CFI	GFI	TLI	IFI	RMSEA
Six-factors model	815.517	338	2.413	0.953	0.904	0.947	0.953	0.051
Four-factors model	3023.756	344	8.790	0.736	0.607	0.710	0.737	0.120
Three-factors model1	4522.961	347	13.034	0.589	0.466	0.552	0.590	0.149
Three-factors model2	3653.382	347	10.530	0.664	0.571	0.634	0.665	0.133
Single-factor model	6258.570	350	17.882	0.418	0.415	0.372	0.420	0.177

### Descriptive Statistics and Correlation Analysis

The mean and standard deviations of each variable as well as the Pearson’s correlation coefficient for these variables are shown in Table 4. The analysis demonstrated that the transformational leadership was significantly and positively related to employees’ thriving at work. Additionally, the transformational leadership was significantly related to challenge-hindrance stressors and challenge-hindrance stressors were also significantly related to employees’ thriving at work. Therefore, it was found to be suitable for further model testing.

**TABLE 4:** Descriptive statistics and correlation analysis.

Variable	1	2	3	4	5	6	7	8	9	10
(1) Gender	1									
(2) Age	0.041	1								
(3) Education	–0.027	−0.171**	1							
(4) Working years	–0.031	0.253**	−0.208**	1						
(5) Job rank	−0.112**	0.126**	−0.125**	0.260**	1					
(1) Transformational leadership style	0.030	–0.073	0.125**	–0.011	0.150**	1				
(2) Challenge stressor	0.052	0.054	0.077	–0.049	0.229**	0.391**	1			
(3) Hindrance stressor	0.212**	0.122**	–0.032	–0.084	−0.121**	−0.262**	0.202**	1		
(4) Employees’ thriving at work	−0.116**	–0.070	0.095*	0.050	0.226**	0.523**	0.294**	−0.364**	1	
(5) Supervisory developmental feedback	–0.003	–0.062	0.003	–0.030	0.115**	0.591**	0.261**	−0.214**	0.460**	1
Mean	1.583	1.716	2.897	1.998	1.542	4.150	3.460	2.991	4.442	4.367
*SD*	0.494	0.623	0.742	0.899	0.786	0.884	1.049	1.147	0.743	0.942

**p < 0.01; *p < 0.05; +*p* < 0.1.

### Common Method Variance Analysis

This study employed two methods to test the severity of homogeneity of variance. According to Harman’s single factor test, the first-factor variance interpretation rate without rotation was 36.938%, which does not account for half of the total variation interpretation. This result indicated the common method variance (CMV) of the data was within the acceptable range (Podsakoff et al., 2000). Additionally, this study employed a method of adding a non-measurable method factor to compare the changes in the model fit after adding the latent variable. Results of the analysis indicated that, after controlling for the common method factors, the fit of the model was not significantly improved (△χ^2^ = 48.86, △df = 26, △χ^2^/df = 1.88). The test results of the above two methods demonstrated the homogeneity of variance in this study was not serious and, therefore, does not impact the reliability of the research conclusions.

### Hypothesis Testing

#### Main and Mediating Effects Analysis

**FIGURE 3:**
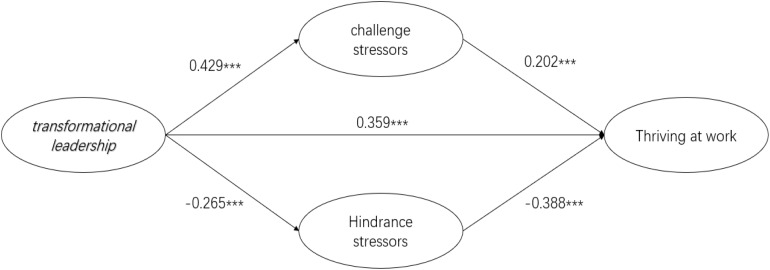
Model path analysis. ***p < 0.001; **p < 0.01; and *p < 0.05.

Using structural equation model, we analyzed the relationship between transformational leadership and employees’ thriving at work, and tested the mediating role of challenge and hindrance stressors. The fitting of the path model was as follows: χ^2^/df = 3.600, CFI = 0.916, GFI = 0.859, TLI = 0.907, IFI = 0.916, and RMSEA = 0.069. This result indicates the fitting effect of the structural equation model generally meets the requirements, and the model sufficiently reflects the objective case of the sample data. The standardized path of the structural model is shown in Figure 3. We can find that the standardized path coefficients of the structural model. There are 5 paths among the variables, all of which are significant at *p* < 0.001. These statistical analysis results provide a basis for the testing and discussion of research hypotheses.

Hypotheses 1, 2, and 3 propose the *relationship between* transformative leadership style and employees’ thriving at work, and challenge and hindrance stressors. Transformative leadership had a direct positive *relationship* with challenge stressors (β = 0.429, *p* < 0.001) and, therefore, Hypothesis 1 is empirically supported. However, transformational leadership had a direct negative *relationship* with hindrance stress (β = −0.265, *p* < 0.001). This result diverges from the original hypothesis of this study and, therefore, Hypothesis 2 is not empirically supported. Transformational leadership has a *relationship* with employees’ thriving at work (β = 0.395, *p* < 0.001) and, thus, Hypothesis 3 is empirically supported. Hypotheses 4 and 5 proposed the *relationship* of challenge and hindrance stressors on employees’ thriving at work. Challenge stressors were found to have a direct positive *relationship* on employees’ thriving at work (β = 0.202, *p* < 0.001) and, therefore, Hypothesis 4 is empirically supported. On the other hand, hindrance stressors had a direct negative *relationship* on employees’ thriving at work (β = −0.338, *p* < 0.001) and, thus, Hypothesis 5 is empirically supported.

Hypotheses 6 and 7 proposed the mediating role of challenge and hindrance stressors. Using model path analysis, challenge stressors were found to play a partial mediating role between transformational leadership and employees’ thriving at work. As can be seen from Figure 3, the total effect of transformative leadership on thriving at work is 0.571, which includes the direct effect of transformative leadership on thriving at work (0.395) and the indirect effects generated by challenge and hindrance stressors [0.429 × 0.202 + (−0.265) × (−0.338) = 0.176].

In addition, challenge stressors have a significant mediating effect (the mediating effect is 0.429 × 0.202 = 0.087) in the relationship between transformative leadership and employees’ thriving at work. At the same time, although judging from the results of the standardized path analysis, hindrance stressors have a significant mediating effect in the relationship between transformative leadership style and employees’ thriving at work the mediating effect is[(−0.265) × (−0.338) = 0.089], However, because the transformative leadership style has a negative effect on hindrance stressors, which is inconsistent with the assumptions in this article, then Hypothesis 7 has not been empirically supported.

#### Moderating Effect Analysis

In this article, the method of hierarchical regression is used to test H8, H9, H10 and H11. The regression results are presented in Table 5. Model 1 is a benchmark model that contains only control variables, while model 2 adds challenge stressors and supervisor developmental feedback on the basis of model 1, and model 3 adds the interactive items of challenge stressors and supervisor developmental feedback on the basis of model 2. Model 4 adds hindrance stressors and supervisor developmental feedback on the basis of model 1, and model 5 adds hindrance stressors and supervisor developmental feedback on the basis of model 4. The VIF of the above regression models are between 1.084 and 1.540, both less than 10. Therefore, the research model does not have multi-collinearity problems and the analysis results are reliable.

**TABLE 5:** Moderating effect analysis.

Variable	Thriving at work				
	Model 1	Model 2	Model 3	Model 4	Model 5
Gender	−0.082 +	−0.100*	−0.091*	–0.040	–0.037
Age	−0.084 +	−0.068 +	−0.060*	–0.023	–0.027
Education	0.114**	0.098*	0.095*	0.106**	0.100**
Working years	0.032	0.069 +	0.066 +	0.027	0.027
Job rank	0.234***	0.134**	0.131**	0.156***	0.155***
Challenge stressors		0.164***	0.069		
Hindrance stressors				−0.245***	−0.248***
Supervisor developmental feedback		0.399***	429^∗∗∗^	0.388***	0.384***
Challenge Stressors × supervisor developmental feedback			0.184***		
Hindrance Stressors × supervisor developmental feedback					0.045
R^2^	0.072	0.281	0.306	0.312	0.312
*F*	9.396***	31.271***	30.769***	35.982***	31.702***
△R^2^		0.210	0.025	0.240	0.002
△F		79.119***	19.622***	94.279***	1.508

***p < 0.001; **p < 0.01; *p < 0.05; +*p* < 0.1.

**FIGURE 4:**
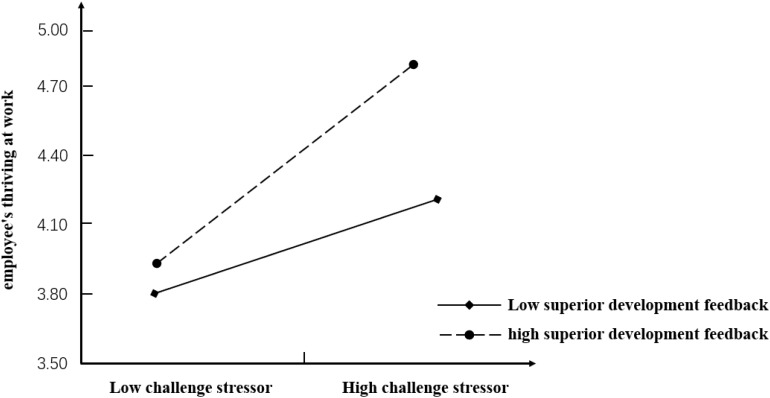
Moderating effect of supervisor developmental feedback on the relationship between challenge stressors and employees’ thriving at work.

Hypothesis 8 proposed that supervisor developmental feedback positively moderates the relationship between challenge stressors and employees’ thriving at work. As seen from Table 5, comparing with model 2, model 3 was improved significantly (△*R*^2^ = 0.025, △*F* = 19.622^∗∗∗^). The standardized regression coefficient of challenge stressors and the supervisor developmental feedback interaction item in Model 3 was found to be 0.215 (*p* < 0.001) and, thus, was statistically significant. To assess whether the moderating effect was consistent with the hypothesis, the relationship between challenge stressors and employees’ thriving at work is plotted in Figure 4. We took the mean value of supervisor developmental feedback, which was added or subtracted by one standard deviation, as the standard of classification. Supervisor developmental feedback can be classified into two types: high-level and low-level developmental feedback. The results of the simple slope test show that with high-level supervisor developmental feedback, there is a significant positive correlation between challenge stressors and employees’ thriving at work (*B* = 0.207, *SE* = 0.032, *p* < 0.001). However, with low-level supervisor developmental feedback, the positive correlation between challenge stressors and thriving at work is not significant (*B* = −0.083, *SE* = 0.055, *p* > 0.1). Therefore, Hypothesis 8 is supported.

Hypothesis 9 proposed that supervisor developmental feedback negatively moderates the relationship between hindrance stressors and employees’ thriving at work. As seen from Table 5, comparing with model 4, model 5 was not improved significantly (△*R*^2^ = 0.002, △*F* = 1.508). The normalized regression coefficient of the interaction between hindrance stressors and supervisor developmental feedback in Model 5 was 0.045 (*p* > 0.1) and, therefore, was not statistically significant. Therefore, Hypothesis 9 is not supported for this study. Consequently, Hypothesis 11 also does not hold true.

#### Moderated Mediating Effect Analysis

Hypothesis 10 proposed that supervisor developmental feedback positively moderates the mediating role of challenge stressors on the relationship between transformational leadership and employees’ thriving at work. We performed the regression of the dependent variable on the independent and moderating variable and showed the moderated mediating effect in Table 6. As shown in Table 4, we find that in Model 5, it was demonstrated that β = 0.360, *p* < 0.001. Specifically, transformational leadership was found to play a significantly positive role in employees’ thriving at work. Second, we performed a regression of the mediator variable on the independent and regulatory variable. In Model 3, it was demonstrated that β = 0.330, *p* < 0.001. Specifically, transformational leadership was found to play a significantly positive role in challenge stressors. Third, in Model 6, we performed a regression of the dependent variable on the independent variable as well as regression of the mediator variable on the regulatory variable. We found the normalized coefficient of the mediator variable (challenge stress) was 0.085 and, therefore, reached a significant level (*p* < 0.05). Additionally, the coefficient of the independent variable was found to be significantly lower than that for Model 4. Given the abovementioned findings, we can see that the mediating effect of challenge stressors was statistically significant. Finally, in Model 7, we performed a regression of the dependent variable on the independent variable, moderate variable, mediator variable, product of the moderate variable and mediator variable. We found that when the product of the moderate variable and the mediator variable enters the regression model, the normalization coefficient of the product of supervisor developmental feedback and challenge stressors was 0.188 and *p* < 0.001. This result illustrates that the moderated mediating effect was statistically significant and, therefore, Hypothesis 10 is supported.

**TABLE 6:** Moderated mediating effect analysis.

Variable	Challenge Stressors			Thriving at work			
	Model 1	Model 2	Model 3	Model 4	Model 5	Model 6	Model 7
Gender	0.079 +	0.059	0.060	−0.110**	−0.105**	−0.111**	−0.101**
Age	0.062	0.088*	0.089*	–0.048	–0.043	–0.050	–0.042
Education	0.100*	0.052	0.055	0.047	0.065 +	0.061 +	0.058
Working years	−0.113*	−0.109**	−0.107*	0.037	0.047	0.056	0.053
Job rank	0.272***	0.206***	0.204***	0.142***	0.136***	0.118**	0.115**
Transformational leadership		0.357***	0.330***	0.496***	0.360***	0.332***	0.335***
Supervisor developmental feedback			0.044		0.230***	0.226***	0.256***
Challenge stressors						0.085*	–0.013
Challenge Stressors × supervisor developmental feedback							0.188***
R^2^	0.072	0.195	0.194	0.306	0.340	0.344	0.370
F	9.683***	22.794***	19.654***	40.484***	40.738***	36.485***	36.252***
△R^2^		0.121	0.001		0.034	0.006	0.026
△F		81.112***	0.850		27.801***	4.725*	22.572***

***p < 0.001; **p < 0.01; *p < 0.05; +*p* < 0.1.

**FIGURE 5:**
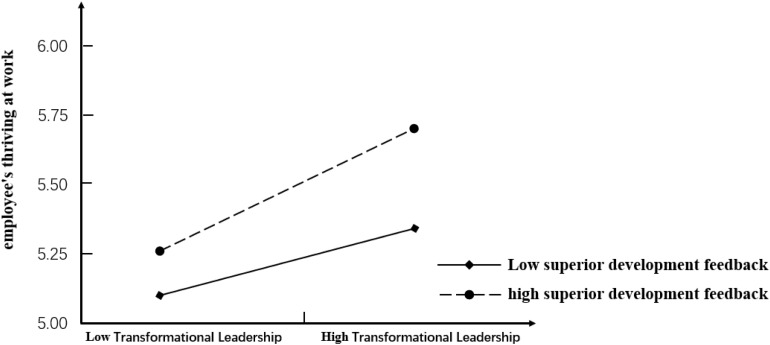
Moderating effect of supervisor developmental feedback on the relationship between transformational leadership and employees’ thriving at work.

To further identify the mediating effect of challenge stressors, supervisor developmental feedback can also be classified into two types: high-level and low-level developmental feedback. The moderating effect of supervisor developmental feedback on the mediating effect is shown in Figure 5. We can find that when challenge stressors is used as a mediator variable, the slope of the line of the higher-level developmental feedback is greater compared with the slope of the line of the lower-level developmental feedback. Specifically, with high-level supervisor developmental feedback, the mediating effect of challenge stressors is strong and positive (Effect = 0.062, *SE* = 0.014). However, with low-level supervisor developmental feedback, the mediating effect of challenge stressors is negative (Effect = −0.067, *SE* = 0.029).

#### The Interaction of Challenge Stressors and Hindrance Stressors

In order to further reveal the relationship between challenge-hindrance stressors and thriving at work, this article adds a model that contains the main effects and interactions of two stressors on thriving at work. Table 7 shows the relationship between challenge-hindrance stressors and thriving at work. In particular, model 1 is a benchmark model that contains only control variables. Model 2 adds independent variable (challenge stressors and hindrance stressors) on the basis of model 1, and finally model 3 adds a product term of challenge and hindrance stressors. From model 2, we can see that there is a positive correlation between challenge stressors and thriving at work (β = 0.355, *p* < 0.001), while there is a negative correlation between hindrance stressors and thriving at work (β = −0.407, *p* < 0.001), and this result supports Hypothesis 4 and Hypothesis 5.

**TABLE 7:** The interaction of challenge stressors and hindrance stressors.

Variable	Thriving at work		
	Model 1	Model 2	Model 3
Gender	−0.082 +	–0.032	–0.035
Age	−0.084 +	–0.047	–0.054
Education	0.114**	0.064 +	0.049
Working years	0.032	0.032	0.037
Job rank	0.234***	0.098**	0.094*
Challenge stressors		0.355***	0.366***
Hindrance stressors		−0.407***	−0.418***
Challenge stressors × Hindrance stressors			0.108**
R2	0.027	0.280	0.290
F	9.363***	31.017***	28.604***
△R^2^		0.208	0.011
△F		78.302***	8.618**

***p < 0.001; **p < 0.01; *p < 0.05; +*p* < 0.1.

In addition, the results of model 3 show that the product of challenge stressors and hindrance stressors is positively correlated with thriving at work (β = −0.108, *p* < 0.01). The results of the simple slope test show that with high hindrance stressors, there is a strong positive correlation between challenge stressors and thriving at work (Effect = 0.347, *SE* = 0.036, *p* < 0.001). However, with low hindrance stressors, the positive correlation between challenge stressors and thriving at work is weaker (*B* = 0.209, *SE* = 0.034, *p* < 0.001). In addition, with high challenge stressors, the negative correlation between hindrance stressors and thriving at work is weak (Effect = −0.231, *SE* = 0.030, *p* < 0.001). With low challenge stressors, the negative correlation between hindrance stressors and thriving at work is strong (*B* = −0.356, *SE* = 0.030, *p* < 0.001).

**FIGURE 6:**
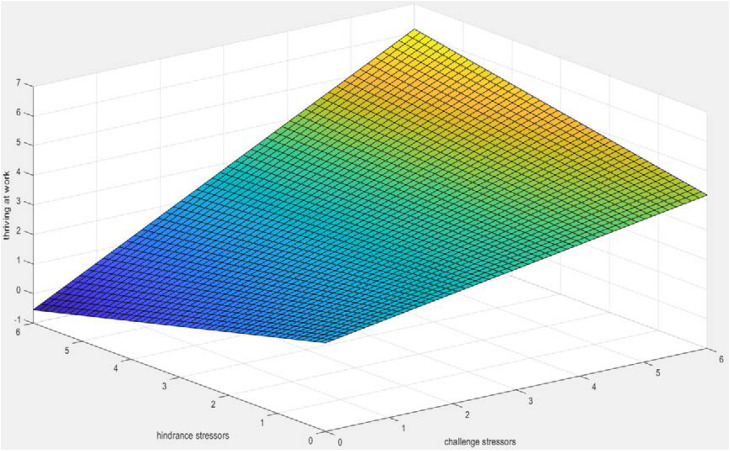
The impact of the interaction of challenging and hindrance stressors on thriving at work.

Although past empirical studies have shown that challenge-hindrance stressors can have very different effects on a series of work-related performance variables such as employees’ attitudes, internal motivations, job inputs, and job performance, so challenge and hindrance stressors can have a completely opposite effect on the sense of work (Podsakoff et al., 2007; Nahrgang et al., 2011; Tadić et al., 2015). However, the interaction between challenge and hindrance stressors can promote employees’ thriving at work. In order to more intuitively observe the relationship between challenge stressors, hindrance stressors and thriving at work, we used MATLAB software to draw three-dimensional graphics. As shown in Figure 6, when the challenge stressors level is low, there is a negative correlation between hindrance stressors and thriving at work, but when the challenge stressors level is high, there is a clear positive correlation between hindrance stressors and thriving at work. And no matter whether the level of hindrance stressors is high or low, challenge stressors always has a positive correlation with thriving at work. When both challenge and hindrance stressors are at a high level, employees will also show a higher level of thriving at work.

## Discussion and Conclusion

### Discussion

The current study found that there is a positive correlation between transformational leadership and employees’ thriving at work, as well as between transformational leadership and challenge stressors. However, there is a negative correlation between transformational leadership and hindrance stressors. Challenge stressors have a significant positive correlation with employees’ thriving at work and hindrance stressors have a significant and negative correlation with employees’ thriving at work. Furthermore, challenge and hindrance stressors play a mediating role in the relationship between transformational leadership and employees’ thriving at work. Additionally, supervisor developmental feedback positively moderates the relationship between challenge stressors and employees’ thriving at work. Supervisor developmental feedback also positively moderates the mediating effect of challenge stressors on the relationship between transformational leadership and employees’ thriving at work. These conclusions contribute to deeper understandings of transformational leadership mechanisms and also contribute to the existing literature on factors associated with employees thriving at work.

First, transformational leadership plays an extremely important role in improving employees’ thriving at work; however, the relationship between these has not been discussed adequately in previous literature. Therefore, the current study incorporated transformational leadership into employees’ thriving at work. Results demonstrate that transformational leadership can effectively promote employee’s thriving at work. More specifically, transformational leadership emphasizes the establishment of an exciting organizational vision for employees, giving employees autonomy in their work, supporting employees to adopt new ideas, experimenting with new methods, and creating a challenging working environment for employees to grow. Therefore, employees who are motivated by targets will form a higher level of internal motivation and, therefore, can experience greater vitality, generate more continuous active learning behavior, and thrive in their workplaces. This conclusion supports the views of Knippenberg and Sitkin (2013), Qu et al. (2015).

Second, this study verifies the moderating effect of supervisor developmental feedback. The study found that when supervisor developmental feedback is higher, challenge stressors have a significant mediating effect on the relationship between transformational leadership and employees’ thriving at work. On the other hand, when supervisor developmental feedback is lower, this mediating effect is not significant. As a supportive resource, supervisor developmental feedback has a significant mitigating effect on the “burnout process” often associated with job requirements. This is consistent with findings produced in the study by Bakker et al. (2003). As external feedback, supervisor developmental feedback also helps to increase employee satisfaction and sense of accomplishment. Transformational leadership, which includes high-performance expectations, empowerment, advocacy, and innovation, is more effective in this work context. Specifically, it helps to enhance employees’ perceptions and experiences of work meaning, self-growth and progress, reduce the consumption of their own resources by challenge stressors and, thus, strengthen the positive relationship between challenge stressors and their thriving at work.

Third, this article illustrates that transformational leadership has a significant negative correlation with hindrance stressors. Although this finding diverges from the prediction made in this study, transformational leadership itself is a complex system and it includes many uncontrollable factors. The reason for this may be that transformational leadership tends to provide resource support to guide employees to focus on the positive side of stress, and to clarify the roles and responsibilities of employees in the process of interacting with them, thereby reducing the many negative stress induced by work ambiguity and uncertainty.

Additionally, the negative moderating effect of supervisor developmental feedback on the relationship between hindrance stressors and employees’ thriving at work was not verified in the current study. This result may be because negative information has a greater impact on individuals than positive information (Skowronski and Carlston, 1989; Kahneman and Tversky, 2013); therefore, even if employees are given supervisor developmental feedback, it is not effective in weakening the negative effect of hindrance stressors. Furthermore, the potential harmfulness associated with hindrance stressors is likely to lead to employees’ lack of intrinsic motivation and, thereby, hinder their personal growth. Compared with the internal subjective evaluation of employees’ self-growth and progress, supervisor developmental feedback is external feedback only. Employees with low mood and a lack of clear targets are significantly less sensitive to external feedback, which makes reducing the negative relationship between hindrance stressors and thriving at work difficult.

The findings of this study also have important implications for the management practices of corporate employees. First, it is important to cultivate a transformative leadership style. Leaders should carefully reflect on their own leadership styles and promote employees’ personal growth and organizational performance through appropriate, positive and effective leadership styles. Second, we should differentiate between positive and negative stressors among employees. Leaders should actively create challenge stressors for employees, such as expanding their scope of work, enhancing job responsibilities, improving perceptions of the meaning of work, and stimulating internal motivation. Some hindrance stressors, such as role blur, role conflicts, and bureaucratic procedures, should be avoided as much as possible. Third, the value of supervisor developmental feedback should be carefully considered. Leaders should provide timely feedback and valuable information to employees based on their actual work situation while trying to avoid the hard requirements which must be met for employees’ specific work tasks as well as placing too much emphasis on standards and expectations. This in turns helps to avoid employees’ excessive perceptions of work stress, stimulate their work vitality to increase their internal need for self-value realization, and also enables them to achieve personal growth, all of which ultimately lead to the collective development of individuals and organizations.

### Theoretical Implication

In line with the development trend of leadership style theory and positive organizational behavior and occupational health psychology, this article explores the mechanism and boundary conditions of transformational leadership on employees’ thriving at work. The main theoretical contributions are as follows: first, based on the logical relationship between social information processing theory, stress-cognitive interaction theory and self-determination theory, this article expounds the transmission mechanism of the transformational leadership related to employees’ thriving at work. Effective intervention of employees’ vitality and learning experience at work provides a possible analytical idea. This study introduces the transformational leadership that has a broad impact on the shaping of job situation characteristics, into the research framework of thriving at work, and also helps to expand the research on antecedent variables of thriving at work.

Secondly, in this article, the transformational leadership and thriving at work are established through challenge and hindrance stressors. The transformational leadership can be related to thriving at work through the perception of challenging and hindrancing stressors. The transformational leadership can directly affect the psychological state of employees, or indirectly affect the psychological state of employees through their perception and evaluation of the characteristics of the work situation. This discovery not only helps to deepen the understanding of the mechanism of transformational leadership, but also opens the black box of the relationship between transformational leadership and employees’ thriving at work.

Thirdly, this article integrates the employees’ subjective experience—thriving at work and external feedback— supervisor developmental feedback into a research framework, which helps to in-depth and comprehensively analyze the employees’ psychological state and behavioral response from the inside and outside. We discuss the regulatory role of supervisor developmental feedback on the relationship between transformational leadership and employees’ thriving at work, and provide empirical evidence for leaders to use supervisor developmental feedback in daily management activities, and also enrich the existing literature for supervisor developmental feedback.

### Practical Implication

The conclusion of this article shows that the transformational leadership has a significant effect on regulating employees’ perception of stress in the workplace and enhancing their experience of working vigorousness. The transformational leadership can stimulate the identity of the followers through the leader’s own charm, strengthen the interactive communication between employees by creating a good atmosphere, be able to focus on personality and activate employee creativity. Therefore, this article recommends that leaders deepen their understanding of transformational leadership s and improve their leadership capabilities. On the basis of insight into the internal and external environment, they plan the future development of the enterprise through the collection and processing of valuable information to enhance employee self-realization, evoke employees’ higher-level needs, improve employee cohesion, enhance employee cooperation, and enable employees to work together to achieve their goals.

Secondly, leaders need to distinguish between two different types of stress in daily management activities and take targeted measures. By choosing appropriate leadership styles and methods, they can create work situations that can make employees feel moderately challenge stressors. And they should reduce as far as possible the sensitive factors contained in the work situation that will trigger the employees’ hindrance stressors perception. However, because challenge stressors can also make employees more enthusiastic about their work and help employees play a greater value in their positions, the point of stress management is not to eliminate various stress.

Finally, as a key means for senior leaders, providing feedback to motivate employees has an important driving role in guiding employees to produce positive work behaviors. Senior leaders should provide timely feedback to employees based on their actual work conditions, try to avoid making hard requirements that must be met for their specific work methods, and do not emphasize standards and expectations too much. They should provide work-related information to make continuous progress, avoid employees from generating excessive stress perception, increase work vitality to evoke employees’ internal needs for self-worth realization, enable employees to obtain personal continuous growth.

## Limitation

The current study has several limitations which should be addressed. First, we collected the cross-sectional data in this article, and only discuss the correlation among the variables. The dynamic relationship cannot be examined rigorously by analyzing cross-sectional data. Second, when we study the relationship between transformational leadership and employees’ thriving at work, we don’t consider the employees’ individual characteristics. For example, some employees may be more sensitive than others. It may be interesting to analyze the relationship between transformational leadership and challenge-hindrance stressors with consideration of employees’ individual characteristics. Third, we only used the questionnaire survey method in this article. The laboratory experiment method and field experiment method can be used to study the mediating roles of challenge-hindrance stressors in future.

## Data Availability Statement

The datasets generated for this study are available on request to the corresponding author.

## Ethics Statement

This study was approved by the Ethical Committee of School of Business Administration of Huaqiao University. We introduced our research purpose, goals, and plans to each participant and asked their permission to participate in this research. The participants can withdraw from the study at any time without penalization. We obtained written informed consent from all participants before data collection. In order to ensure ethical standards in the data collection process, we adhere to the relevant ethics required for quantitative research. Specifically, in order to find research participants, we visited companies in Fuzhou, Xiamen, and Quanzhou, and asked the employees of the company to fill out the questionnaire and collect the questionnaire. Before the questionnaire was issued, we communicated with the relevant person in charge of the company and obtained their consent. With their consent, we only distributed the questionnaire to the employees. In other words, we started the investigation after obtaining the consent of the participants and the manager of the company. The questionnaire in offline surveys is mainly issued on the Wenjuanxing app. The sample sources include Fujian, Zhejiang, Shanghai, Guangdong, and Jiangsu. Because online surveys are anonymous, they protect the privacy of participants.

## Author Contributions

CL, HH, and BL jointly built the model, conceived the research ideas, discussed the results, and revised the manuscript. The contribution of CL was mainly in theory, and the article was critically revised in the later period. JX’s contributions are mainly in methodological issues and data analysis. BL and JX were jointly responsible for the review of the article, ensuring that the research conducts the investigation in an appropriate manner to ensure the accuracy and completeness of the research. All authors contributed to the article and approved the submitted version.

## Conflict of Interest

The authors declare that the research was conducted in the absence of any commercial or financial relationships that could be construed as a potential conflict of interest.
